# Peptidomics-based identification of endogenous free peptides from *Ophiocordyceps sinensis* and functional validation of immunomodulatory peptide WALGT

**DOI:** 10.3389/fphar.2026.1748787

**Published:** 2026-05-01

**Authors:** Wei Wang, Yuanping Liu, Yunjie Hu, Huan Yang, Mengjiao Zhang, Congcong Li, Yuting Mu, Lilian Zhao, Yuping Wan, Yun Deng

**Affiliations:** 1 Key Laboratory of Standardization of Chinese Medicine, Ministry of Education, School of Pharmacy, Chengdu University of Traditional Chinese Medicine, Chengdu, China; 2 Key Laboratory of Monitoring and Assessment on Novel Food Raw Materials, State Administration for Market Regulation, Chengdu Institute of Food Inspection, Chengdu, China; 3 Yibin Vocational College of Medicine and Health, Yibin, China

**Keywords:** endogenous free peptides, immunomodulation, macrophage proliferation, Ophiocordyceps sinensis, peptidomics, WALGT

## Abstract

Ophiocordyceps sinensis (formerly known as *Cordyceps sinensis*), commonly referred to as “dongchongxiacao” in Chinese, is a valuable traditional Chinese medicine whose bioactive small molecules form the essential material basis for its therapeutic effects. Nonetheless, endogenous free peptides, as a class of potentially important active constituents, have remained relatively underexplored due to the high cost of *Ophiocordyceps sinensis*. In this study, peptidomics technology combined with the confidence scoring mechanism of MaxQuant analytic software (Andromeda score) was employed. Peptides for which each amino acid residue corresponds to a unique fragment ion signal in the tandem mass spectrometry (MS/MS) spectrum were designated as high-confidence identified peptides. High-confidence peptide samples were synthesized using solid-phase technology and subsequently verified using reverse comparative analysis of MS/MS fragmentation spectra under identical mass spectrometric conditions. This approach further ensured the accuracy of sequence analysis for endogenous free peptides in *Ophiocordyceps sinensis*. Our method requires merely milligram quantities of *Ophiocordyceps sinensis* samples for the detection of endogenous free peptides, which is a notable methodological improvement over the traditional activity-guided stepwise separation methods with high sample consumption used in previous peptide studies. Different from previous reports focusing on artificially enzymatically hydrolyzed peptides/peptide-like compounds, we identified native endogenous free peptides independent of proteolysis in *Ophiocordyceps sinensis*, and using RAW 264.7 cell culture, the peptide WALGT was confirmed to exert immunomodulatory effects via promoting macrophage proliferation. The proliferative effect was additionally confirmed using scratch test, colony formation assay, and proliferation factor analysis. Using a mouse immunosuppression model, the findings indicated that WALGT treatment markedly reinstated monocyte/macrophage and white blood cell (WBC) counts and the expression levels of immunological factors (IgG, IgM) in CTX-exposed mice relative to the model group, confirming the ability to enhance the immunological function of immunosuppressed mice. This study aims to provide a novel research strategy for analyzing endogenous free bioactive peptides using only milligram-level samples.

## 1 Introduction

*Ophiocordyceps sinensis* (syn. *Cordyceps sinensis*), commonly referred to as “dongchongxiacao” in Chinese, is a traditional Chinese medicine that has garnered considerable interest owing to its distinctive pharmacological properties and rarity. Presently, investigations concerning *Ophiocordyceps sinensis* predominantly emphasize nucleosides, polysaccharides, and several other constituents (Wang et al., 2022). *Ophiocordyceps sinensis*, as an animal-derived therapeutic substance, is abundant in proteins. The significance of proteins as a crucial source of amino acids has been thoroughly established. Recent years have seen the acknowledgment that proteins perform numerous supplementary activities in the body via bioactive peptides. Bioactive peptides are specific protein fragments that positively impact bodily activities or circumstances and may eventually affect health (Splith and Neundorf, 2011; Zheng et al., 2011; Wang et al., 2021). As a category of potentially significant active components, they vary in size from 2 to 30 amino acid residues, and their pharmacological significance warrants more exploration (Xiao et al., 2022). While many bioactive peptides can be directly sourced from nature, the majority require hydrolysis for their release from proteins (Cai et al., 2017; Sun et al., 2017; Ano et al., 2019; Jakubczyk et al., 2020). Relevant reports on *Ophiocordyceps sinensis* peptides to date have focused on this type of hydrolyzed peptides/peptide-like compounds obtained by artificial enzymatic hydrolysis (Xu et al., 2023; Li B. et al., 2024). In addition, there is another class of bioactive peptides that are independent of proteolysis for their generation and exist in a free state within organisms. And the systematic research on this type of native endogenous free peptides in *Ophiocordyceps sinensis* has not been reported yet. Recent studies have focused on the predicted peptides, proteome, and metabolome of Ophiocordyceps sinensis, but native endogenous free peptides have not been experimentally identified and validated (Zhang et al., 2020; Tong and Guo, 2022; Wang et al., 2024; Jia et al., 2025).

The systematic investigation and functional analysis of these endogenous free peptides are still rather inadequate. The peptide constituents of traditional Chinese remedies are exceedingly intricate and highly polar, complicating the separation and purification of individual monomers. This renders the conventional activity-guided stepwise separation technique ineffective, significantly impeding the research advancement of free endogenous peptides in animal-derived therapeutic materials (Li et al., 2018; 2019; Wang et al., 2019; Ji et al., 2022). The advancement of peptidomics technology has rendered sequencing via ultra-high performance liquid chromatography-tandem mass spectrometry (UPLC-MS/MS) an increasingly significant tool for identifying undiscovered peptide-based pharmacologic agents (Abd El-Aziz et al., 2020; Tracz et al., 2021). Nonetheless, in practical detection, the existence of absent peaks and contaminated peaks in mass spectrometry disrupts the precise alignment of fragment ions, thus diminishing the accuracy of amino acid sequence prediction in peptidomics (Klaproth-Andrade et al., 2024; McDonnell et al., 2022). To address the technical limitations of low identification accuracy for trace peptides and high sample consumption associated with traditional methods, we classified peptide sequences as reliable in this study if they yielded an Andromeda score above 100, using the built-in confidence scoring function of MaxQuant software. Simultaneously, by MS/MS spectrum validation, peptides with each amino acid residue corresponding to distinct fragment ion peaks were selected as the final dependable identification outcomes (Medzihradszky and Chalkley, 2015; Huang et al., 2016). Pure peptide products were synthesized using solid-phase methods and subsequently analyzed with HPLC-MS/MS technology. By replicating the same chromatographic-mass spectrometric conditions employed earlier, the precision of endogenous peptide identification from *Ophiocordyceps sinensis* was further validated through a reverse comparison of the software analysis results, primary molecular mass, and MS/MS fragment ion data between the predicted sequences and the synthetic peptides.

*Ophiocordyceps sinensis*, a historic and esteemed tonic herb, exhibits immunostimulatory effects, which constitute one of its fundamental pharmacological properties validated throughout millennia of use (Olatunji et al., 2018). Contemporary pharmacological research suggests that the influence of *Ophiocordyceps sinensis* on the immune system is not merely a “enhancement” of immunity, but rather the manifestation of a distinct “immunomodulatory” activity (Wei et al., 2021). The active components can influence the immune system via multiple targets and pathways, notably by activating the phagocytic and cytotoxic functions of macrophages and natural killer cells, enhancing the proliferation and differentiation of T and B lymphocytes, and modulating the expression of various cytokines (Qi et al., 2020; Li S. et al., 2024). Nonetheless, for an extended period, research has predominantly concentrated on active constituents such as cordycepin, cordyceps polysaccharides, and adenosine, whereas investigations into the immunostimulatory effects of endogenous free peptides from *Ophiocordyceps sinensis* remain comparatively limited (Krishna et al., 2024; Yang et al., 2025). There is now widespread agreement that bioactive peptides exhibit distinct immunostimulatory and immunomodulatory properties. Furthermore, owing to their low molecular weight and uncomplicated structure, these small-molecule peptides are more readily absorbed and utilized by the body, and can also directly interact with the surface receptors of immune cells to exercise their effects. This study posits that the endogenous free peptides in *Ophiocordyceps sinensis* may be a crucial active component contributing to its immunomodulatory activity (Hemmati et al., 2024; Lim et al., 2025).

This study proposes a working hypothesis based on the premise that *Ophiocordyceps sinensis* forms a typical host-pathogen parasitic complex and existing literature reports on insect larval innate immune defense responses to fungal infection (Li et al., 2020; Wang et al., 2022): during the resistance of host larvae to *Ophiocordyceps sinensis* fungal infection, host larvae may accumulate specific defensive peptides, which is tentatively hypothesized to constitute a potential material basis for the immunomodulatory activities of *Ophiocordyceps sinensis*. Significantly, these bioactive peptides can be produced without protein hydrolysis and exist as endogenous free peptides inside biological systems.

## 2 Materials and methods

### 2.1 Extraction of endogenous peptides

A 100 mg sample of *Ophiocordyceps sinensis* was weighed and pulverized into a fine powder, followed by the addition of ultrapure water at a solid-to-liquid ratio of 1:10 (g:mL). The mixture underwent ultrasonic extraction for 30 min while maintained in an ice bath. Following extraction, centrifugation was conducted at 5,000 × g and 4 °C for 10 min, and the supernatant was obtained as the crude extract of *Ophiocordyceps sinensis* peptides. The supernatant was transferred to an ultrafiltration tube with a 10 kDa molecular weight cutoff, then subjected to centrifugation at 1,000 × g and 4 °C for 10 min to exclude macromolecular contaminants, including proteins and nucleic acids. The permeate was gathered and desalted with a C18 column to eliminate small-molecule contaminants, resulting in the *Ophiocordyceps sinensis* peptide solution. The peptide solution was lyophilized to get *Ophiocordyceps sinensis* peptide powder.

### 2.2 Identification of peptides by peptidomics sequencing

The peptides were solubilized in 0.1% formic acid in water for liquid mass spectrometry analysis. The Orbitrap Exploris 480, in conjunction with the Easy-nLC 1200 liquid chromatography system including a capillary column (75 μm ID, 15 cm length, filled with 2 μm nanoViper C18) from Thermo Fisher Scientific, United States, was employed to separate peptides at a flow rate of 300 nL/min. Mobile phase A consisted of 0.1% formic acid, while mobile phase B comprised 80% acetonitrile in an aqueous solution of 0.1% formic acid. The gradient for liquid phase separation is as follows: 0–8 min, 3%–5% B; 8–66 min, 5%–20% B; 66–77 min, 20%–38% B; 77–85 min, 38%–95% B; and 85–90 min, 95% B, utilizing a sample volume of 500 ng.

Data were acquired using the DDA mode of Orbitrap Exactive mass spectrometry. The parameters for mass spectrometry are as follows: NSI ionization source spray, voltage set at 2.4 kV, scan duration of 60 min, positive ion scan mode, ion transport capillary temperature of 300 °C, full scan resolution of 60,000, scan range of 400–1800 *m/z*; the secondary mass spectrometry scan range begins at 110 *m/z*, with a resolution of 15,000 and a maximum ion injection time of 22 ms. The HCD fragmentation mode was employed with a normalized collision energy (NCE) of 27. To enhance the effective utilization of mass spectrometry, the automatic gain control (AGC) was configured to 5E4, the signal threshold was established at 5,000 ions/s, and the dynamic exclusion time for the repeated acquisition of ions was set to 45 s.

Mass spectral raw data were processed using MaxQuant software (v2.6.2.0) with the integrated Andromeda search engine. A database-dependent targeted searching strategy was applied for peptide identification. The reference amino acid sequence database adopted in this study consisted of the reviewed protein sequences of *Ophiocordyceps sinensis* downloaded from the UniProt database, and common contaminant protein sequences provided within MaxQuant. Peptide identifications with an Andromeda score >100 were regarded as high-confidence results and used for subsequent data analysis.

### 2.3 Cell viability by Ophiocordyceps sinensis peptide solution and synthetic peptides

RAW 264.7 cells were purchased from Zhejiang Meisen Cell Technology. All cell lines were respectively cultured in DMEM media containing 10% fetal bovine serum (FBS) and 1% penicillin-streptomycin. Cells were incubated at 37 °C in a humidified incubator containing 5% CO_2_.

Cell proliferation inhibition was monitored by CCK-8 assay. Exponentially growing RAW 264.7 cells were seeded into 96-well plates at a density of 5 × 10^3^ cells/mL and attached for 24 h. Then the *Ophiocordyceps sinensis* peptide solutions at different concentrations (0.1, 0.15, 0.2, 0.25, 0.3, 0.35, and 0.4 mg/mL) or synthetic peptides (FQDPL, MLSPV, WALGT, VFDPT, APPPP, LVLGL, YSPPHYPSPW, GPPPP, APLSL, LNLP, WLINQ) at concentrations of 6.25, 12.5, 25, 50, 100 μg/mL were added to the corresponding wells. After further incubation for 48 h, 100 μL of serum-free medium containing 10% CCK-8 reagent was added to each well, and the plates were incubated again in the 37 °C constant-temperature incubator for additional 1 h. Subsequently, the absorbance value at a wavelength of 450 nm was measured.

### 2.4 Colony formation

We implemented colony formation experiments to investigate the ability of WALGT (selected based on preliminary CCK-8 dose-response assays (Figure 3)) to promote the colony formation. After overnight cell adherence, the medium containing peptide WALGT (final concentration: 50 μg/mL, selected based on preliminary CCK-8 dose-response assays (Figure 3) showing this concentration elicited a robust, statistically significant (P < 0.001) proliferative effect on RAW 264.7 cells with clear biological relevance) was added to the wells of the experimental group, and incubation was continued for 48 h. Then, cells were incubated in a serum-free medium for 5 days and fixed in 4% para-formaldehyde, stained with 0.1% crystal violet solution. The total number of colonies was observed under microscopy. ImageJ software was used for quantitative analysis of the number and size of formed colonies.

### 2.5 Wound healing assay

By evaluating the migratory capacity, we aimed to investigate the ability of the WALGT to promote cell proliferation. After the RAW 264.7 cells were cultured to full confluence, a wound was created by scraping the cell monolayer with 200 μL sterile pipette tips. Reference images were specified on the plate and a time 0 h image was obtained. Cells were cultured with indicated doses of WALGT (50 μg/mL, the optimal concentration selected from CCK-8 dose-response data (Figure 3) for its significant pro-proliferative effect on RAW 264.7 cells) for 48 h. The second image was acquired in the matched original region. All images were compared to a reference image and ImageJ software was used to measure the wound healing area and calculate the wound healing rate.

### 2.6 Reverse transcription quantitative PCR (RT-qPCR)

Total RNA was isolated utilizing the Column Animal Tissue Total RNA Extraction and Purification Kit. The primer sequences are presented in Table 1. The RT-qPCR reaction conditions were set according to the manufacturer’s guidelines: reverse transcription at 45 °C for 5 min; pre-denaturation at 94 °C for 30 s; followed by 40 cycles of denaturation at 94 °C for 5 s, annealing at 59 °C for 15 s, and extension at 72 °C for 10 s. A melting curve analysis was subsequently performed to assess non-specific amplification and eliminate potential interference, including primer dimers. All experiments was repeated at least 3 times. Relative gene expression was determined using the 2^-ΔΔCt technique with GAPDH as the internal reference, and the specific primers employed in this investigation are detailed in Table 2. The experimental results were documented and subjected to statistical analysis utilizing GraphPad Prism software (V9.5.0).

**TABLE 1:** Sequence of primers used for RT-qPCR.

Gene	Primer sequences (5′–3′)
GAPDH	F: 5′-GAAGGTCGGTGTGAACGGAT-3′ R: 5′-CCCATTTGATGTTAGCGGGAT-3′
Ki-67	F: 5′-CAGACCCAGAGCCTGACAAC-3′ R: 5′-GGCGGTCTTGTTGATGTTGT-3′
Cyclin D	F: 5′-CGTGGCGTGTAAGATGAAGG-3′ R: 5′-GGAAGCGGTCCAGGTAGTTC-3′

**TABLE 2:** The reaction system of RT-qPCR.

Ingredient	Volume
Green one-step qPCR SuperMix (2X)	5 μL
Primer Mix (10 μM each)	0.4 μL
Green one-step RT/RI enzyme mix	0.2 μL
RNA template	1 μL
RNase-free water	3 μL
Total volume	10 μL

### 2.7 Proteomics analysis using label-free nano-LC-MS/MS

The medium with peptide WALGT (final concentration: 50 μg/mL, chosen based on preliminary CCK-8 dose-response assays (Figure 3) demonstrating this concentration induced a robust and statistically significant proliferative response in RAW 264.7 cells, ensuring detectable molecular changes for proteomic analysis) was introduced to the wells of the experimental group. Three biological replicate samples of total protein extraction were conducted following the guidelines of the Total Protein Extraction (TPE) Kit (Sangon Biotech Co., Ltd.). The protein concentration in the supernatant was quantified with the BCA assay kit. Thereafter, the protein sample underwent protease digestion via the filter-aided sample preparation (FASP) method (Wiśniewski et al., 2009). Trypsin digestion was performed overnight at 37 °C. The digested peptides were desalted using Pierce™ C18 Tips, after which the desalted peptide samples were transferred to centrifuge tubes and subjected to freeze-drying in a vacuum freeze dryer. The freeze-dried peptides were utilized for later mass spectrometry analysis.

Data were acquired using the DIA mode of Orbitrap Exactive mass spectrometry. The parameters for mass spectrometry are as follows: NSI ionization source spray, voltage set at 2.4 kV, scan duration of 60 min, positive ion scan mode, ion transport capillary temperature of 300 °C, full scan resolution of 120,000, scan range of 380–980 *m/z*; the secondary mass spectrometry scan range begins at 120 *m/z*, with a resolution of 30,000 and a maximum ion injection time of 54 ms. The HCD fragmentation mode was employed with a normalized collision energy (NCE) of 30. To enhance the effective utilization of mass spectrometry, the automatic gain control (AGC) was configured to 5E4, the signal threshold was established at 5,000 ions/s, and the dynamic exclusion time for the repeated acquisition of ions was set to 40 s. The liquid phase parameters are the same as those in 2.3. The mass spectrometry proteomics data have been deposited to the ProteomeXchange Consortium (https://proteomecentral.proteomexchange.org) via the iProX partner repository (Ma et al., 2019; Chen et al., 2022) with the dataset identifier PXD076647.

Gene Ontology (GO) enrichment analysis, including biological process (BP), cellular component (CC), and molecular function (MF), and Kyoto Encyclopedia of Genes and Genomes (KEGG) pathway enrichment analysis were performed using the DAVID online tool (https://davidbioinformatics.nih.gov/home.jsp). Terms with P < 0.05 were considered significantly enriched. The enrichment factor was calculated as the ratio of the number of differentially expressed proteins assigned to a pathway to the total number of proteins annotated in that pathway.

### 2.8 Rescue CTX-induced immunosuppressed phenotypes

After 7 days of acclimatization feeding, specific pathogen-free (SPF) male BALB/c mice (6–8 weeks old, weighing 18–22 g) were randomly divided into six groups (n = 6 per group): blank control group, model group, positive control group, and low-, medium-, and high-dose treatment groups. The control group and model group were administered normal saline via oral gavage. The positive control group received Jinshuibao Capsule (50 mg/kg) via gavage, which was selected as the positive control for its close relevance to the study: it is a clinically validated Cordyceps militaris-derived preparation, homologous to *Ophiocordyceps sinensis* with consistent core immunomodulatory efficacy (Olatunji et al., 2018; Wei et al., 2021), a commonly used positive control in Cordyceps-related immunomodulation research, and its oral administration route is identical to that of peptide WALGT, ensuring experimental comparability and eliminating route-related bias. The treatment groups were administered the WALGT peptide at low (25 mg/kg), medium (50 mg/kg), and high (100 mg/kg) doses for a duration of 14 consecutive days. Between days 12 and 14, except for the blank control group, all mice were intraperitoneally injected with cyclophosphamide at a dose of 50 mg/kg once daily for three consecutive days (Days 12, 13 and 14 after grouping) to establish the immunosuppressive model. Following the therapy period, full blood count assays and assessments of IgG and IgM levels were conducted.

## 3 Result

### 3.1 Identification and profiling of peptides from Ophiocordyceps sinensis by UPLC-MS/MS

**FIGURE 1:**
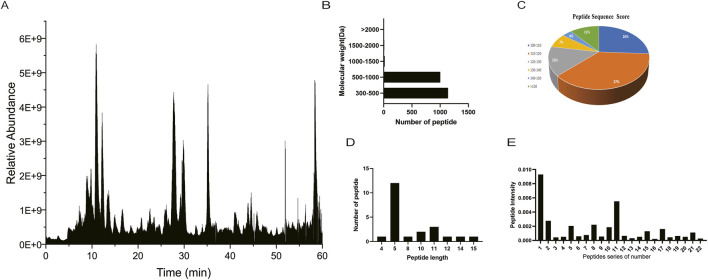
**(A)** Total ion chromatogram of *Ophiocordyceps sinensis* peptides. **(B)** Molecular weight. **(C)** Peptide sequence scores. **(D)** Length distribution of the 22 polypeptides. **(E)** Intensities of the 22 polypeptides.

Figure 1A displays the total ion chromatogram (TIC) of peptides from *Ophiocordyceps sinensis*. The peak ion intensity of the peptides reached approximately E9 (10^9^), which met the requirements for sensitivity and stability in mass spectrometry analysis. High-Collision Dissociation (HCD) was applied to acquire MS/MS spectra, allowing the detection of characteristic b- and y-ions from peptides separated by nano-UPLC. Mass spectrometry data were processed using MaxQuant (v2.6.2.0) with the Andromeda search engine. Precursor mass tolerances were set to 20 ppm for the first search and 4.5 ppm for the main search (Group-specific parameters > Instrument). For high-resolution Orbitrap HCD MS/MS spectra, the fragment mass tolerance was internally fixed at 0.02 Da by the software (default for Orbitrap data in MaxQuant 2.6.2.0). Peptide identification was controlled by peptide-level FDR ≤0.01, protein-level FDR ≤1, and an Andromeda score >100 was applied as a stringent filter for high-confidence peptide matches. The key parameters are listed in Table 3. In total, 2170 peptides were identified, with molecular weights mainly distributed between 300 and 1000 Da (Figure 1B). Using an Andromeda score >100 as the filtering threshold (Bartlett et al., 2018; Xiang et al., 2022), 22 peptides were considered reliably identified in this study (Figure 1C). These peptides were dominated by pentapeptides (Figure 1D). The relative intensities of these 22 peptides were further determined (Figure 1E).

**TABLE 3:** Parameters for peptidomics sequencing.

Parameters	Setting
Digestion	Unspecific
Fasta file	Uniprotkb_Ophiocordyceps_sinensis
First search peptide tolerance	20 ppm
Main search peptide tolerance	4.5 ppm
Fragment mass tolerance	0.02 Da
Min.peptide length	4
PSM FDR	1%
Protein FDR	100%

Finally, the amino acid sequences, lengths, molecular weights, confidence scores, and predicted activity scores of the 22 peptides are summarized in Table 4.

**TABLE 4:** The peptides with a score greater than 100.

No	Sequence	Length	Mass	Score	Peptide ranker
1	YVERPNPR	8	1029.536	166.17	0.245588
2	FQDPL	5	618.3013	160.00	0.854974
3	GPPGLPVPPPHP	12	1160.634	159.14	0.828948
4	PAPAPAPAPPP	11	981.5284	149.94	0.794127
5	MLSPV	5	545.2883	138.59	0.323863
6	VNLSL	5	544.3221	127.53	0.159623
7	NELTL	5	588.3119	126.60	0.108403
8	WALGT	5	546.2802	123.86	0.605080
9	YPPPDHGYPPPGQG	14	1477.663	123.79	0.731754
10	AAKKAAAAAK	11	970.5924	121.09	0.171131
11	VFDPT	5	577.2748	118.11	0.447653
12	YPPPDHGYPPP	11	1235.561	117.93	0.786518
13	QLPINRPVVPIHNNN	15	1723.948	116.30	0.396527
14	APPPP	5	477.2587	111.84	0.930420
15	LVLGL	5	513.3526	111.84	0.312076
16	VGLTV	5	487.3006	108.73	0.075323
17	TLDDPKPPPK	10	1106.597	108.43	0.436662
18	YSPPHYPSPW	10	1229.551	106.04	0.886715
19	GPPPP	5	463.2431	104.08	0.950298
20	APLSL	5	499.3006	102.87	0.510751
21	LNLP	4	455.2744	102.45	0.445204
22	WLINQ	5	672.3595	102.40	0.413116

### 3.2 Identification and validation of peptide sequences by MS/MS and synthetic peptide confirmation

**FIGURE 2:**
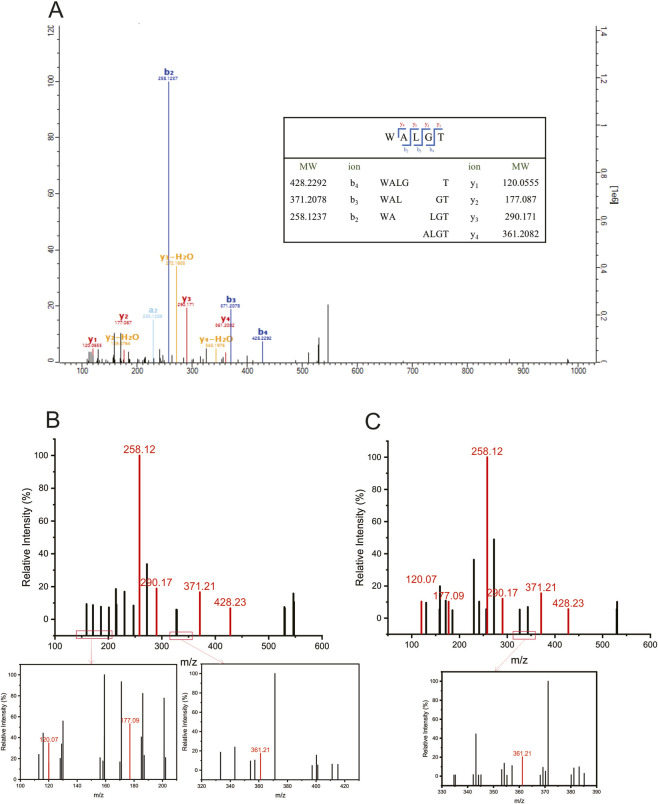
**(A)** The secondary spectrum of WALGT calculated by MaxQuant software, and the b and y ions of WALGT. **(B,C)** MS/MS spectra of the identified WALGT peptide from natural *Ophiocordyceps sinensis* peptide extracts **(B)** and the chemically synthesized WALGT standard **(C)**.

Additionally, MS/MS spectra of peptides with scores exceeding 100 were carefully verified. Manual validation of mass spectra is required during peptide sequence identification. A peptide sequence is considered accurate and reliable only when continuous b/y ion series covering the entire peptide backbone (with key cleavage sites from N- to C-terminus) are observed in the spectrum, and the amino acid residue sequence can be unambiguously determined without conflicting fragment ions. If only a limited number of fragment ions are matched or no continuous ion series can be established, generally only the amino acid composition can be confirmed, whereas the specific amino acid sequence order cannot be accurately assigned. Finally, 11 peptides with unique amino acid sequences were identified, and these are highlighted in bold in the Table 4. For example, the peptide with the sequence WALGT was confirmed by its y ion series at *m/z* 120.0555, 177.0870, 290.1710, and 361.2082, and b ion series at *m/z* 258.1237, 371.2078, and 428.2292 (Figure 2A). Fragment ions corresponding to each amino acid residue of WALGT were distinctly detected, confirming the sequence without ambiguity. To verify the accuracy of identification, the candidate peptides were chemically synthesized using solid-phase synthesis and re-evaluated under the same HPLC-MS/MS conditions as in the discovery phase. The sequences were unequivocally confirmed by comparing the mass spectrometric profiles (including both precursor and fragment ions) between the endogenous peptides from *Ophiocordyceps sinensis* and their synthetic peptides. The identical fragment ions were accurately detected in both the natural peptide from *Ophiocordyceps sinensis* and the synthetic peptide. For the sequence WALGT, the characteristic y-ion series appeared at *m/z* 120.0555, 177.0870, 290.1710, and 361.2082, and the b-ion series at *m/z* 258.1237, 371.2078, and 428.2292 (Figures 2B,C).

### 3.3 Proliferative activity of Ophiocordyceps sinensis peptide WALGT on RAW 264.7 cells

**FIGURE 3:**
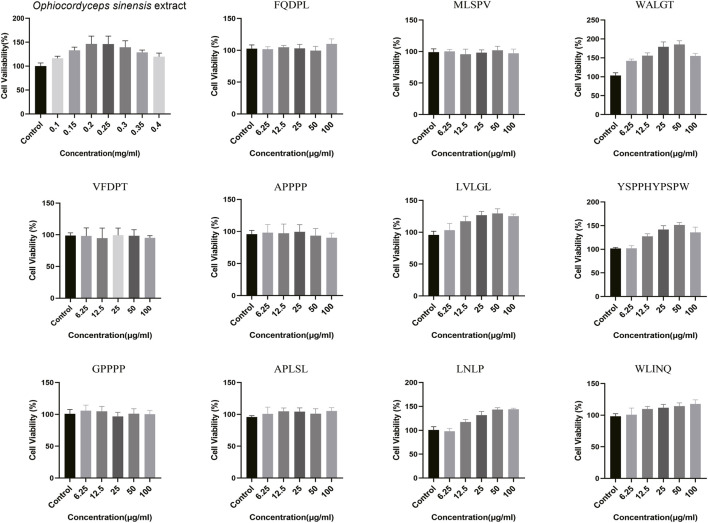
Effect of *Ophiocordyceps sinensis* extract and synthetic peptides on cell viability. Cells were treated with *Ophiocordyceps sinensis* extract (0.1, 0.15, 0.2, 0.25, 0.3, 0.35, 0.4 mg/mL) or synthetic peptides (FQDPL, MLSPV, WALGT, VFDPT, APPPP, LVLGL, YSPPHYPSPW, GPPPP, APLSL, LNLP, WLINQ) at concentrations of 6.25, 12.5, 25, 50, 100 μg/mL for 48 h. Cell viability was determined by CCK-8 assay and expressed as percentage relative to the untreated control group (set as 100%).

First, we examined the proliferative effects of *Ophiocordyceps sinensis* peptides extract on RAW 264.7 cells. The CCK-8 assay was used to evaluate the effects of peptide solutions at different concentrations (0.1, 0.15, 0.2, 0.25, 0.3, 0.35, and 0.4 mg/mL) on macrophage proliferation. As shown in Figure 3 (first plot), compared with the blank control group (0 mg/mL), treatment with 0.2–0.3 mg/mL peptides for 48 h significantly increased cell proliferative activity, and cell viability was enhanced in a concentration-dependent manner.

To identify the key active peptide responsible for the observed proliferative activity, we first performed solid-phase synthesis of all 11 peptides with unique and validated amino acid sequences (screened via Andromeda score >100 and manual MS/MS spectrum validation, shown in bold in Table 4). A preliminary CCK-8 proliferation assay was then conducted on RAW 264.7 macrophages to evaluate the pro-proliferative potential of each synthetic peptide. Among these 11 peptides, WALGT exhibited the most significant and statistically robust pro-proliferative effect on RAW 264.7 cells (P < 0.001), thus being selected as the target peptide for subsequent in-depth functional validation and mechanistic investigation (Figure 3).

**FIGURE 4:**
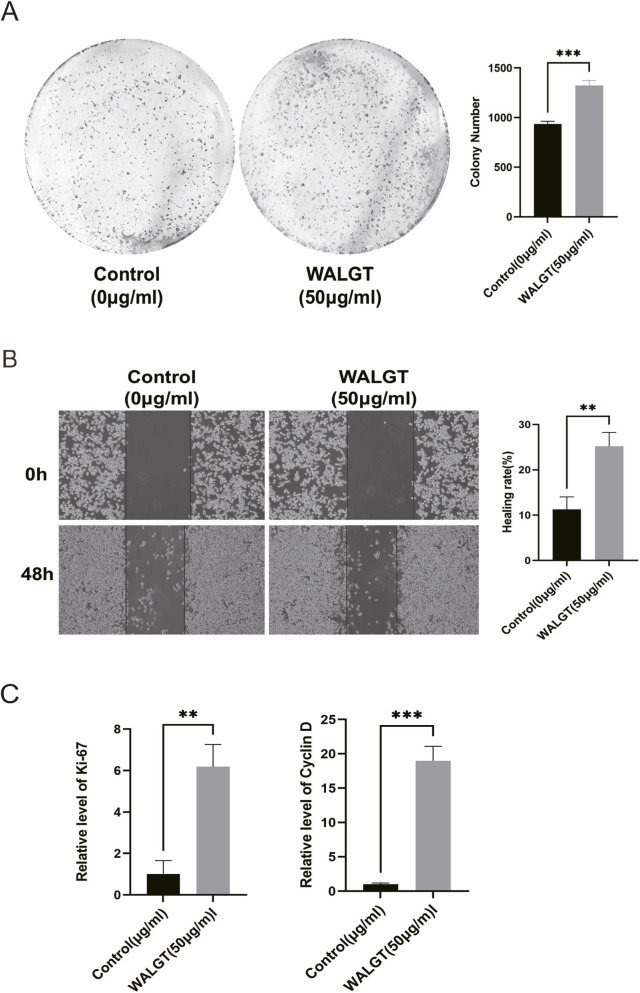
WALGT promotes the proliferation of RAW 264.7 cells. **(A)** Colony forming assays. RAW 264.7 cells were exposed to WALGT (50 μg/mL) for 48 h and colonies were stained using crystal violet 2 weeks later. The rate was analyzed by ImageJ. **(B)** Wound-healing assay. RAW 264.7 cells migration was observed at the indicated concentration (50 μg/mL) and healing rate was analyzed by ImageJ. **(C)** RT-qPCR of proliferation factors. Each experiment was repeated at least 3 times. *P < 0.05, * *P < 0.01, * **P < 0.001, vs. the control group.

Colony formation assays showed that WALGT treatment remarkably increased the number of cell colonies, confirming its ability to promote cell proliferation (Figure 4A). Wound healing assays revealed that WALGT significantly enhanced the migration of RAW 264.7 cells toward the scratched area (Figure 4B). Furthermore, quantitative real-time PCR (qPCR) analysis demonstrated that WALGT treatment significantly upregulated the mRNA expression of the cell proliferation marker Ki-67 and the cell cycle regulatory gene Cyclin D, suggesting that WALGT promotes the proliferation of RAW 264.7 cells at the transcriptional level (Figure 4C).

Collectively, these results indicate that WALGT can effectively promote the proliferation and migration of RAW 264.7 cells *in vitro*. This immunostimulatory effect *in vitro* prompted us to further investigate whether WALGT exerts broader immunomodulatory activity in an immunosuppressed animal model.

### 3.4 Quantitative proteomic analysis of WALGT-Treated RAW 264.7 cells

For protein identification and quantification, raw mass spectrometry data were processed using DIA-NN (version 1.8.1) against the *Mus musculus* database (UniProt ID: 10090). The identification results were filtered using the following criteria: at least one unique peptide per protein and false discovery rate (FDR) ≤ 1%. Relative protein abundances were determined using label-free quantification. Quantitative data were median-normalized, and missing values were imputed with half the minimum observed value using an online analysis platform (https://www.bioladder.cn/web/#/toolSet). Differentially expressed proteins (DEPs) were identified using T-test with thresholds of |log2FC (fold change) | > 1 and P < 0.05. Multiple testing correction was performed using the Benjamini–Hochberg false discovery rate (FDR) method. An adjusted P < 0.05 was considered statistically significant.

**FIGURE 5:**
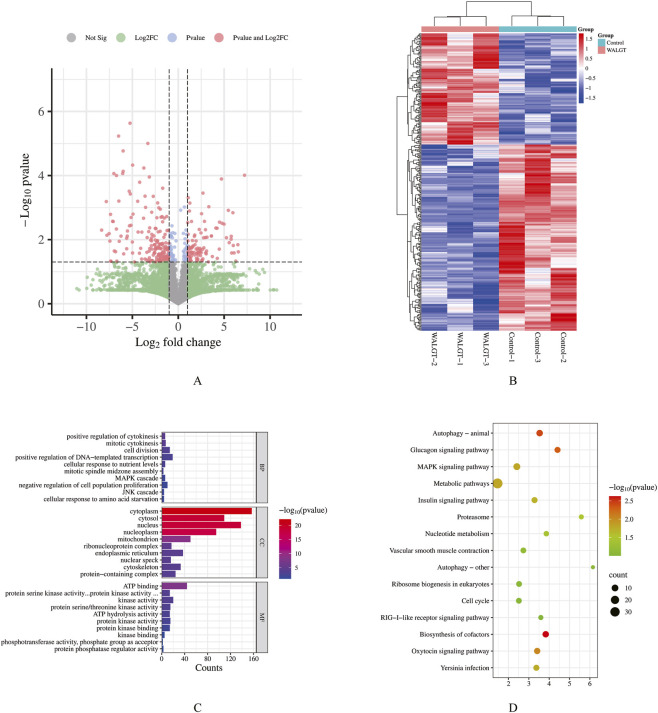
Differentially expressed genes/proteins analysis in WALGT-treated cells. **(A)** Volcano plot of differential expression (|Log_2_FC| ≥ 2, p < 0.01). **(B)** Clustering heatmap of differentially expressed genes/proteins. **(C)** GO enrichment analysis (BP, CC, MF) of differentially expressed genes/proteins. **(D)** KEGG pathway enrichment bubble plot of differentially expressed genes/proteins.

To investigate proteomic changes induced by WALGT, comparative analysis was performed between WALGT-treated and control groups. The volcano plot (Figure 5A) showed that numerous DEPs, among which several displayed extremely high significance (−log10(P) > 4) and large fold changes (|log2FC| > 5). Hierarchical clustering heatmap analysis (Figure 5B) further confirmed that samples were distinctly divided into WALGT and control clusters, indicating good experimental reproducibility. DEPs showed two opposite expression patterns: one cluster was upregulated in the WALGT group, while the other was highly expressed in the control group. These results demonstrated that WALGT systematically remodels the protein expression profile and regulates specific biological pathways in RAW 264.7 cells.

To explore the biological functions of DEPs, Gene Ontology (GO) and Kyoto Encyclopedia of Genes and Genomes (KEGG) pathway enrichment analyses were performed. GO enrichment revealed that DEPs were mainly enriched in biological processes including cell proliferation, cell division, and mitotic cell cycle; molecular functions such as protein kinase activity, ATP binding, and microtubule binding; and cellular components related to cell division, including spindle, centrosome, and microtubule cytoskeleton (Figure 5C). KEGG analysis further showed significant enrichment in canonical proliferation-related pathways, including cell cycle, DNA replication, MAPK, PI3K-Akt, and mTOR signaling pathways, as well as core metabolic pathways and nucleotide biosynthesis which provide essential energy and biosynthetic precursors to support macrophage proliferation and immune activation (Figure 5D).

Collectively, the identified DEPs were primarily involved in regulating cell cycle progression, maintaining mitotic spindle assembly, activating downstream proliferative signaling, and supporting cellular metabolism and biosynthesis. These results suggest that WALGT may promote cell proliferation through multiple molecular mechanisms by modulating these DEPs, providing a functional basis for understanding its regulatory effects on the observed cellular phenotypes.

### 3.5 WALGT attenuates cyclophosphamide-induced immunosuppression in mice

**FIGURE 6:**
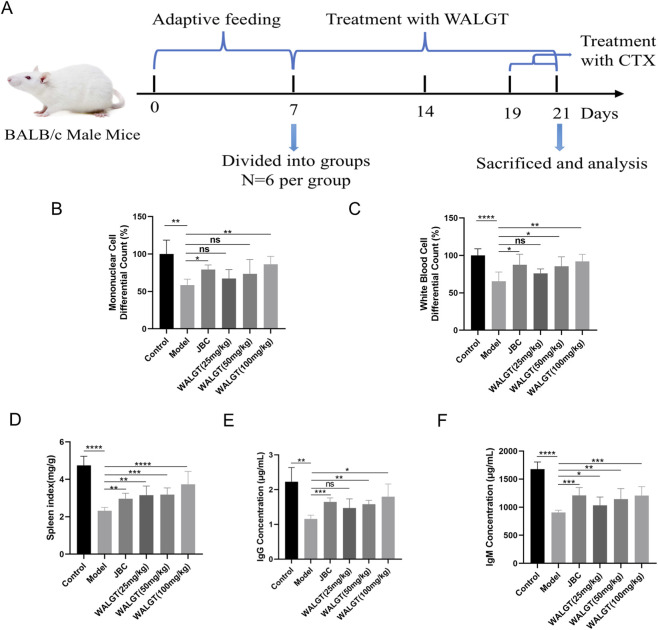
WALGT mitigates CTX-induced immunosuppression. **(A)** Schematic of mouse model establishment and WALGT administration. **(B)** Monocyte/macrophage counts. **(C)** White blood cell (WBC) counts. **(D)** Spleen index of mice. **(E)** Immunoglobulin G (IgG) levels. **(F)** Immunoglobulin M (IgM) levels.

We established a mouse model of cyclophosphamide (CTX)-induced immunosuppression to evaluate the immunorestorative effects of the peptide WALGT (Figure 6A). On the penultimate day of the experiment, retro-orbital blood samples were collected for hematological analysis. As shown in Figures 6B,C, WALGT treatment significantly restored monocyte/macrophage and white blood cell (WBC) counts in CTX-exposed mice compared with the model group. WALGT also dose-dependently increased the spleen index (Figure 6D). Moreover, the levels of immunoglobulin G (IgG) and immunoglobulin M (IgM), key markers of humoral immunity, were significantly elevated in WALGT-treated mice relative to the model group (Figures 6E,F).

These results collectively demonstrate that WALGT alleviates CTX-induced immunosuppression by enhancing both innate and adaptive immune responses.

## 4 Conclusion

This study comprehensively identified a series of endogenous free peptides in *Ophiocordyceps sinensis* using a mass spectrometry-based peptidomic method, utilizing only milligram-scale samples. A stringent methodology was implemented, incorporating high-confidence software scoring and MS/MS fragment ion validation on an individual basis. Furthermore, solid-phase synthesis and reverse mass spectrometry alignment were employed to substantially enhance the accuracy of peptide sequence identification. This not only addresses the limitations of conventional activity-guided isolation techniques in peptide research but also offers an effective and dependable framework for investigating trace bioactive constituents in valuable therapeutic substances.

This is the first confirmation of native proteolysis-independent endogenous free peptides in *Ophiocordyceps sinensis*, distinct from previously reported artificially enzymatically hydrolyzed peptides/peptide-like compounds. Among 11 synthetic peptides with validated unique sequences, WALGT was selected for in-depth validation due to its most significant pro-proliferative effect on RAW 264.7 macrophages (P < 0.001) in preliminary CCK-8 assays. Functional validation showed WALGT markedly enhances the proliferation, migration and colony-forming capacity of RAW 264.7 macrophages *in vitro* and upregulates proliferation markers (Ki-67, Cyclin D). Mechanistically, WALGT exerts immunomodulatory effects via a metabolism-driven proliferation network: it regulates core metabolic pathways and nucleotide biosynthesis to support cell proliferation and immune activation for cell cycle progression and PI3K-Akt signaling activation, and *in vivo* restores innate/adaptive immunity in CTX-induced immunosuppressed mice by promoting B lymphocyte differentiation and IgG/IgM secretion.

This finding complements existing *Ophiocordyceps sinensis* research: unlike cordycepin and polysaccharides (which act via inflammatory signaling inhibition or macrophage phagocytosis activation), WALGT modulates immunity through metabolic reprogramming, further supporting the multi-component, multi-target characteristic and synergistic immunomodulatory effects of the fungus’s active constituents (nucleosides, polysaccharides, endogenous free peptides). WALGT is identified as a novel immunomodulatory peptide, enriching the understanding of *Ophiocordyceps sinensis*’ medicinal material basis and establishing a rigorous methodology for studying endogenous peptides in valuable medicinal materials.

This study has several limitations. First, RAW 264.7 cells, though widely used, may not fully replicate primary macrophage biology. Second, the CCK-8 assay measures metabolic activity, which could partially confound proliferation readouts. Third, while oral WALGT showed *in vivo* efficacy, its gastrointestinal stability and degradation kinetics were not directly assessed. Addressing these points—through primary cell validation, orthogonal proliferation assays, and pharmacokinetic studies—will be essential to advance WALGT toward clinical or nutraceutical applications. Future research will use thermal proteomics to identify WALGT’s intracellular targets, conduct LC-MS/MS-based pharmacokinetic profiling, and perform peptide combination assays to explore synergistic effects. These studies will comprehensively elucidate WALGT’s immunomodulatory network, laying a solid foundation for developing natural peptide-based immunomodulatory pharmaceuticals and functional foods.

## Data Availability

The mass spectrometry proteomics data have been deposited to the ProteomeXchange Consortium via iProX with dataset identifier PXD076647.
